# IDOL N342S Variant, Atherosclerosis Progression and Cardiovascular Disorders in the Italian General Population

**DOI:** 10.1371/journal.pone.0122414

**Published:** 2015-04-30

**Authors:** Ashish Dhyani, Gianpaolo Tibolla, Andrea Baragetti, Katia Garlaschelli, Fabio Pellegatta, Liliana Grigore, Giuseppe Danilo Norata, Alberico Luigi Catapano

**Affiliations:** 1 Department of Pharmacological and Biomolecular Sciences, University of Milan, Milan, Italy; 2 I.R.C.C.S MultiMedica, Milan, Italy; 3 SISA Center for the Study of Atherosclerosis, Bassini Hospital, Cinisello B., Italy; 4 The Blizard Institute, Centre for Diabetes, Barts and The London School of Medicine & Dentistry, Queen Mary University, London, United Kingdom; University of Lleida, SPAIN

## Abstract

Inducible degrader of the low density lipoprotein receptor (IDOL), is an E3 ubiquitin ligase that negatively modulates low density lipoprotein receptor (LDL-R) expression. Genome-wide association studies (GWAS) indicated that genetic variants in IDOL gene contributes to variation in LDL-C plasma levels and the detailed analysis of a specific locus resulted in the identification of the functional common single nucleotide polymorphism (SNP) rs9370867 (c.G1025A, p.N342S) associates with increased LDL-R degradation and increased LDL-C levels. These findings, however, were not confirmed in two other independent cohorts and no data about the impact of this variant on atherosclerosis progression and cardiovascular risk are available. Aim of this study was to investigate the association between a functional variant in IDOL and atherosclerosis progression in an Italian general population. 1384 subjects enrolled in the PLIC study (Progression of Lesions in the Intima of Carotid) were genotyped by Q-PCR allelic discrimination and the association with anthropometric parameters, plasma lipids and the carotid intima media thickness (cIMT) and the impact on cardiovascular disease (CVD) incidence were investigated. The N342S variant was not associated with changes of the plasma lipid profile among GG, AG or AA carriers, including total cholesterol (249±21, 249±19 and 248±21 mg/dl respectively), LDL-C (158±25, 161±22 and 160±23 mg/dL), cIMT (0.74±0.14, 0.75±0.17 and 0.77±0.15 mm) and CVD incidence. In agreement, the expression of LDLR and the uptake of LDL was similar in macrophages derived from GG and AA carriers. Taken together our findings indicate that the N342S variant does not impact plasma lipid profile and is not associated with atherosclerosis progression and CVD in the general population, suggesting that other variants in the IDOL gene might be functionally linked with cholesterol metabolism.

## Introduction

Elevated levels of circulating low density lipoprotein cholesterol (LDL-C) represent a key factor for cardiovascular disease (CVD) risk [1]. Plasma LDL-C levels are mainly regulated by the production and the clearance of apolipoprotein B (apoB) containing lipoproteins by the liver. A central role in the hepatic LDL-C metabolism is played by the low density lipoprotein receptor (LDLR) that mediates the uptake of LDL in the hepatocytes, thus promoting their clearance [2]. LDLR gene mutations account for the majority of the cases of autosomal dominant hypercholesterolemia (ADH 1), a genetic disease characterized by elevated LDL-C levels and premature CVD death [3]. The LDLR activity is controlled at the transcriptional level by the nuclear translocation of the sterol regulatory element binding protein 2 (SREBP2) [4]. LDL-R expression is also controlled by the pro-protein convertase subtilisin-like kexin type 9 (PCSK9) which binds the LDLR at the plasma membrane of the hepatocytes and induces its degradation in the lysosomes [5]. Circulating PCSK9 is mainly produced by the liver and in analogy with the LDLR is controlled by SREBP2 activity, thus making PCSK9 an interesting target for the development of lipid lowering drugs [6]. In addition to PCSK9, the inducible degrader of the LDLR (IDOL, also known as MYLIP) also controls the LDLR abundance by mediating the ubiquitination of the intracellular tail of the receptor and its lysosomal degradation [7]. In contrast to PCSK9, IDOL expression is ubiquitous and regulated by the oxidized sterols sensitive nuclear receptor liver X receptor (LXR) [8]. Hepatic overexpression of the IDOL gene in mice results in hypercholesterolemia and atherosclerosis development [7,9]. In humans IDOL has been suggested as a candidate gene involved in the modulation of lipoproteins metabolism by genome-wide association studies (GWAS)[10–12]. Further investigation aimed at identifying the IDOL genetic variants responsible for the association generated controversial results. In a Mexican dyslipidemic population fine mapping of the IDOL gene identified the common rs9370867 SNP as the susceptibility variant associated with total cholesterol levels [13]. The rs9370867 SNP encodes the amino-acid substitution N342S, located in the FERM domain of the protein, a critical region involved in the regulation of protein-protein interaction. The presence of a Serine residue (encoded by the G allele) reduces the ability of the IDOL protein to ubiquitinate the LDLR, thus increasing plasma membrane LDLR expression and LDL clearance. This observation was not replicated in two Brazilian cohorts, characterized by mixed ethnicity, where the same variant was not associated with LDL-C levels both the general population and in patients with stable angina [14]. Finally, analysis of IDOL gene variants in the Dutch population showed similar allelic frequencies of the rs9370867 SNP in two cohorts with extremely high and low LDL-C levels [15], further questioning the functional relevance of this variant. In this study we aimed at clarifying the contribution of the rs9370867 SNP in the IDOL gene to plasma cholesterol levels and the association with a preclinical marker of atherosclerosis such as the common carotid artery intima media thickness (cIMT). For this purpose we genotyped a cohort representative of the Italian general population enrolled in the PLIC study (Progression of Lesions in the Intima of Carotid- Caucasian ethnicity)[16–18] and we investigated the association of the rs9370867 SNP with the lipid profile, cIMT, and the incidence of cardiovascular disease (CVD) after a 10 years follow-up. We found no association of plasma lipid profile with cIMT and CVD incidence. Moreover we observed similar LDL uptake capacity in macrophages generated from peripheral blood mononuclear cells from IDOL rs9370867 GG or AA carriers. Taken together our results indicate that other variants, if any, are likely to be responsible for the relationship between plasma LDL-C levels and genetic variation in the IDOL gene evidenced by recent GWAS in Caucasian populations.

## Materials and Methods

### Population sample

A cohort of 2141 subjects attending the Atherosclerosis Center in Bassini Hospital, Department of Pharmacological and Biomolecular Sciences (University of Milan, Italy), were recruited for the PLIC study. The PLIC study was approved by the Ethics Commitee of the University of Study of Milan (approved on 06-02-2001 SEFAP protocollo n°0003/2001), all participants signed a written informed consent. The investigation was performed in accordance with the principles of the Declaration of Helsinki. This project is a study designed to investigate the presence and progression of atherosclerotic lesions and intima media thickness (IMT) in the carotid artery of a large local cohort in relation to the major cardiovascular disease risk factors. Exclusion criteria were use of hypolipidemic drugs, presence of liver or kidney disease, thyroid dysfunction. 1384 subjects gave their signed consent to use their DNA for genetic studies addressing the cardiovascular system. Biochemical and clinical variables were evaluated as previously described [19,20]. High resolution B-mode ultrasonography of carotid Intima-Media Thickness (c-IMT) with a linear ultrasound probe (4.0–13.0 MHz frequency, 14X48 mm footprint, 38 mm field of view) was performed (Vivid S5 GE Healthcare®, Wauwatosa, WI, USA). The determination were performed by a single sonographer, blinded to the subject’s identity (intra‐class correlation = 0.812, n = 30). All the measurements were done off-line using the software provided by the instrument [21]. The protocol involved the common carotid artery (CCA) (30 mm proximal to the carotid bulb), the carotid bulb and internal carotid artery (ICA) at both sides. The intima-media thickness (IMT) was assessed at the far wall as the distance between the interface of the lumen and intima, and the interface between the media and adventitia in a standardized number of points [21,22]. The maximal IMT was recorded and averaged for the left and right sides of the CCA (30 mm proximal to the carotid bulb), the carotid bulb, the ICA. Presence of extra-cardiac atherosclerotic vascular involvement was determined with presence of focal plaques (> 1.3 mm in longitudinal resolution, lateral or medial angle) and/or diffusive mean IMT > 1.3 mm (in longitudinal resolution, lateral or medial angle)[23].

The protocol involved the determination of the Left Ventricular Mass (LVM) assessment. The measurements were performed by two dimensional guided M-mode echocardiography from the parasternal window using an M-mode color-Doppler (Vivid S5 GE Healthcare®, Wauwatosa, WI, USA) (1.4–4.0 MHz frequency, 19.3X27.6 mm footprint wide-band phased array transducer). Left Ventricular Mass (LVM) was calculated with Devereux’s formula, according to guidelines. The definition of cardiovascular events (CVEs) included coronary heart disease (CHD), such as acute myocardial infarction (AMI), acute coronary syndromes, acute and chronic heart failure (New York Heart Association class II and III); peripheral artery disease (PAD) and cerebrovascular events, such as stroke, transient cerebral ischemic attack in the previous 6 months, and other vascular complications (including diabetic foot ulcers). In addition having undergone major surgery was included in this definition (i.e.: carotid thrombo-arterectomy, percutaneous coronary angioplasty, arterial angioplasty or arterial by-pass of the lower limbs, coronary by-pass and amputations). Cardiovascular risk was defined in accordance to the Progetto Cuore individual risk score, for the Italian population as described [23].

### Genotyping

Genomic DNA was extracted from buffy coat samples using the Flexigene DNA kit (Qiagen, Milan, Italy) according to the manufacturer’s instructions. Genotyping for the rs9370867 IDOL SNP (c.G1025A, p.N342S) was performed on 5 μL (10–200 ng of DNA), using a TaqMan allelic discrimination test. Taqman SNP Genotyping assay Code N. C_2461770_10 (Thermo Fisher Scientific, Applied Biosystems, Waltham, MA).

### Peripheral blood mononuclear cells and macrophages cell culture

Peripheral blood mononuclear cells (PBMCs) were collected from 10 patients previously enrolled in the PLIC study (n = 5 carriers of the AA genotype and n = 5 carriers of the GG genotype, the two groups were matched for sex and age) as reported [24]. Briefly blood was diluted 1: 3 in Phosphate Buffered Saline (PBS) (15 mL) then layered onto 4 mL of Ficoll Hipaque (Amersham) and centrifuged at 1500 rpm for 35 min. PBMCs were removed from the interface and washed twice in PBS before being re-suspended in RPMI-1640 medium supplemented with penicillin (50 U/ml), streptomycin (50 μg/ml), L-glutamine (2 mM) and 10% serum AB. PBMCs were counted and 2x10^6^ cells were plated in 6 well plates and incubated for 2 hours in a humidified atmosphere (37°C, 5% CO_2_). Non adherent cells were removed with four rinses of PBS and attached monocytes cultured for 7 days to obtain monocyte-derived macrophages.

### Gene expression analysis

Total RNA was extracted from monocytes-derived macrophages and 1 μg of RNA underwent reverse transcription using the IScript cDNA Synthesis kit (BioRad, Milan, Italy) [24,25]. Three μL of cDNA were amplified by real-time quantitative PCR with 2X MAXIMA SYBR Green/Fluorescein qPCR mastermix (Carlo Erba Reagents, Cornaredo, Italy)[26]. The specificity of the Sybr green fluorescence was tested by plotting fluorescence as a function of temperature to generate a melting curve of the amplicon. Each sample was analyzed in duplicate using the CFX Connect Real Time detection system (BioRad, Milan, Italy). The primers used are the following: *18S*: Fw 5’-CGCAGCTAGGAATAATGGAATAGG-3’, Rw5’- CATGGCCTCAGTTCCGAAA-3’; *LDLR*: Fw 5’- GTGTCACAGCGGCGAATG -3’, Rw 5’- CGCACTCTTTGATGGGTTCA -3’. *IDOL*: Fw 5’- GATAACAGAGACGCACGCATTC -3’, Rw 5’- CCCTTCAAGTCACGGCTATACTG -3’.

### LDL preparation and macrophage LDL uptake assay

LDL (density 1.019–1.063 g/ml) were obtained from freshly isolated human plasma from healthy volunteers by preparative ultracentrifugation in KBr gradient, dialyzed versus PBS containing 0.01% EDTA and sterilized by filtration. Protein content was determined by the colorimetric Lowry assay, using BSA as a standard. We then used a flow-cytometry assay to evaluate LDL uptake by using fluorescently labelled LDL, a widely and robust assay currently used for the investigation of LDLR function [27,28]. 1mg of LDL was labelled overnight at 4°C in 1 mL PBS containing 0,5mg/mL of 3,3′-dioctadecyloxacarbocyanine perchlorate (DiO) (stock solution 5mg/mL in dimethylformamide) (Sigma Aldrich, Milan, Italy). DiO-LDL were then isolated again by ultracentrifugation in KBr gradient and the excess of fluorescent probe and KBr salt was removed by dialysis versus PBS containing 0.01% EDTA. LDL were sterilized by filtration and the protein content determined as previously described. Monocytes-derived macrophages were washed with PBS then incubated with 10μg/mL of DiO-LDL in RPMI without serum

After 2 hours cells were washed three times with PBS, gently scraped off and collected in tubes for flow cytometry analysis. Cells were immediately analyzed using a FACSCalibur flow cytometer (BD Biosciences) and CellQuest software.

### Statistical analysis

Data were analyzed using IBM-SPSS 21.0 for Windows (Chicago, IL, USA). Results are reported as mean ± SD, if not otherwise stated; Grubb’s test was performed to verify presence of outliers data. Group differences among genotypes were determined by using one way Analysis of Variances (ANOVA) test followed by LSD and Bonferroni post-hoc analyses; otherwise Kruskal-Wallis test was then performed to compare not normally distributed variables across the three genotypes. Then the Multivariable Analysis of Variances (MANOVA) was performed to adjust the comparisons with other covariates, if significantly different among genotypes. Student’s t-test for continuous normally-distributed variables (when appropriate) and χ^2^ analysis for categorical variables were performed for the analysis of the alleles. Group differences with P < 0.05 were deemed as statistically significant.

## Results

### General characteristics of subjects from the PLIC population according to the rs9370867 SNP

No deviation from the Hardy-Weinberg equilibrium was observed for the rs9370867 (c.G1025A) SNP. In the PLIC population analyzed (n = 1384) the allelic frequencies were 48.01% and 51.99% for the G and A allele respectively, no significant differences in the genotypes distribution between men (n = 485) and women (n = 899) were observed **(Table 1)**. No major differences were found in the anthropometric and biochemical parameters, such as total cholesterol (TC), LDL-C, high density lipoprotein cholesterol (HDL-C), triglycerides (TG) and glycaemia among carriers of the different genotypes. **(Table 2)**. Previous association of the rs9370867 SNP with cholesterol metabolism were observed in a Mexican dyslipidemic population, characterized by high levels of TC and TG. For this reason we stratified the PLIC population according to TG and TC plasma levels and we repeated the analysis in two sub-groups upper the 75^th^ percentile of TC (mean value 271.2 ± 23.7 mg/dL) and TG (mean value 191 ± 71,1mg/dL). The results were similar compared to the whole population and showed no significant impact of the rs9370867 (c.G1025A) SNP on the lipid profile, **S1A and S1B Table**

**Table 1:** Genotypic [n(%)] and allelic frequencies (%) of the rs9370867 SNP in the PLIC population.

		Genotype			Allele	
	n	GG	GA	AA	G	A
**PLIC**	1384	328(23.7%)	673(48.6%)	383(27.7%)	48.01%	51.99%
**Men**	485	127(26.2%)	230(47.4%)	128(26.4%)	49.9%	50.1%
**Women**	899	201(22.4%)	443(49.3%)	255(28.4%)	47%	53%
χ2	2.621				2.552	
**P-value**	0.454				0.110	

**Table 2:** Anthropometric and biochemical characteristics according to the rs9370867 SNP in subjects from the PLIC population.

	GG	AG	AA	P-value GG vs AA	P-value GG vs GA	P-value AA vs GA
**Age**	65.21 ± 9.97	64.36 ± 1.084	64.90 ± 9.88	0.735	0.342	0.515
**Body mass index (Kg/m2)**	26.8 ± 4.6	26.7 ± 4.3	27.0 ± 4.2	0.756	0.739	0.456
**Total cholesterol (mg/dL)**	209.9 ± 35.9	206.1 ± 34.6	204.7 ± 34.3	0.089	0.172	0.570
**LDL-cholesterol (mg/dL)**	125.2 ± 32.8	123.3 ± 31.9	122.6 ± 31.6	0.356	0.449	0.775
**HDL-cholesterol (mg/dL)**	64.60 ± 18	63.08 ± 16.18	62.64 ± 14.8	0.168	0.249	0.701
**Triglycerides (mg/dL)**	100.5 ± 42.4	98.91 ± 48.0	97.27 ± 45.6	0.391	0.646	0.634
**Apolipoprotein A-I (mg/dL)**	158.2 ± 23.4	156.4 ± 21.0	155.3 ± 20.7	0.133	0.311	0.455
**Apolipoprotein B (mg/dL)**	108.7 ± 22.1	105.6 ± 22.2	107.0 ± 22.2	0.389	0.078	0.380
**SBP (mmHg)**	128 ± 17	129 ± 18	128 ± 18	0.568	0.352	0.749
**DBP (mmHg)**	82.03 ± 9.4	82.37 ± 8.7	82.32 ± 8.8	0.983	0.228	0.195
**Glucose (mg/dL)**	95.38 ±15.89	94.97 ± 17.93	92.94±13.72	0.057	0.762	0.091

### Impact of the rs9370867 SNP for IDOL on carotid atherosclerosis and the prevalence of cardiovascular events

**Fig 1:**
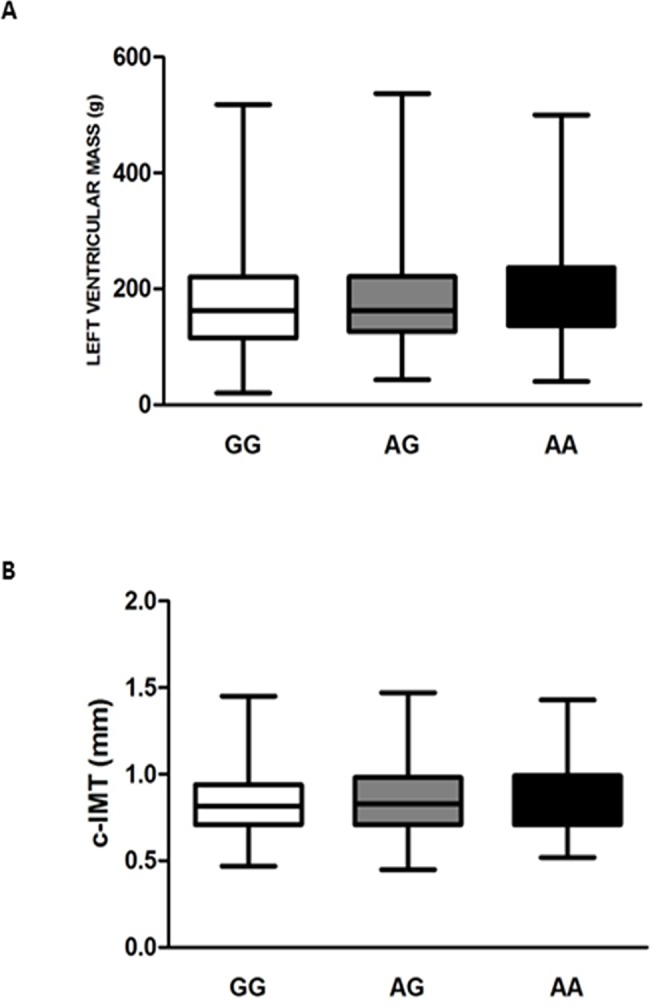
Heart left ventricular mass and carotid intima media thickness of the common carotid arteries in the PLIC population according to the rs9370867 IDOL SNP. Panel A shows the mean ± SD for left ventricular mass for GG, AG or AA carriers. Panel B shows the mean ± SD of carotid intima media thickness of the common carotid arteries (right and left) for GG, AG or AA carriers.

**Fig 2:**
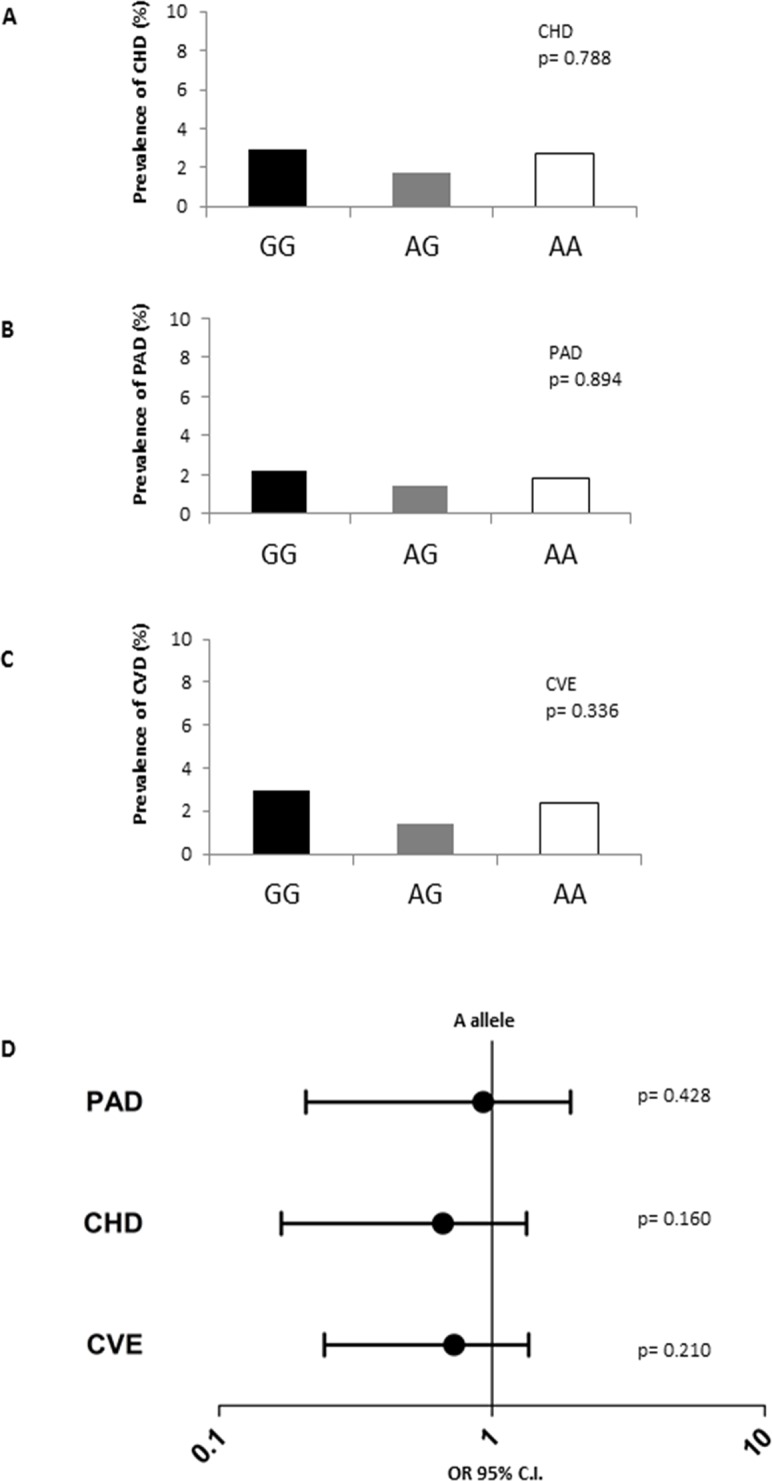
Incidence of coronary heart disease (CHD), peripheral artery disease (PAD) and cardiovascular events (CVE) in the PLIC population according to the rs9370867 SNP. Panels A, B and C shows the incidence (%) of CHD, PAD and CVE for GG, AG or AA carriers of the rs9370867 IDOL SNP. Panel D shows the risk CHD, PAD and CVE for the carriers of A allele of the rs9370867 vs carriers of the G allele. Odds Ratios (OR, the 95% Confidence Interval (C.I.), adjusted for age, gender, lipid profile, systolic blood pressure, glucose levels and therapies) are not statistically significant.

Although no association between the rs9370867 SNP and LDL-C or other lipid variables were observed, this does not exclude the possibility that this functional SNP might affects other pathways associated with cardiovascular disease. Therefore to further investigate the potential role for the rs9370867 SNP, its association with left ventricular mass and intima media thickness of carotid arteries (**Fig 1A and Fig 1B**) and the incidence of coronary heart disease, peripheral artery disease and cardiovascular events (CHD, PAD and CVE) was investigated **(Fig 2A to Fig 2C)**. The results show that the rs9370867 genotype is not associated with any of the parameters investigated. Also when carriers of the A allele both in homozygosis and in heterozygosis were compared to carriers of the G allele, no differences in the odd ratio for the incidence of CHD, PAD or CVE were observed. These data limit the impact of rs9370867 SNP for IDOL on cardiovascular outcome.

### LDLR expression and activity according to the rs9370867 SNP

**Fig 3:**
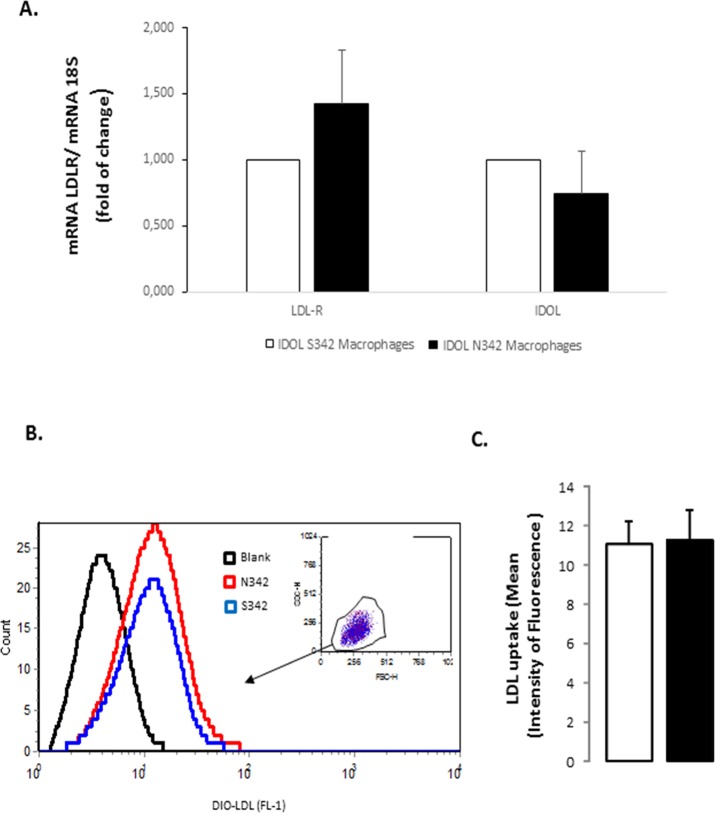
mRNA expression of LDL-R and IDOL and LDL uptake in macrophages from N342 and S342 macrophages. Panel A shows mRNA expression for LDL-receptor and for IDOL in macrophages obtained from N342 and S342 carriers (n = 5 for both genotypes) cultured in complete RPMI medium The results, normalized for the expression of an housekeeping gene (18S), data are shown as mean ± SEM. Next flow cytometry was used to study the LDL uptake in macrophages obtained from N342 or S342 carriers. To determine the LDL uptake, macrophages were incubated with fluorescently labelled LDL (DiO-LDL, 10 μg/mL) for 2 hours at 37°C. Panel B shows representative images for flow cytometry in N342 and S342 macrophages, with the gating strategy of macrophages (inside panel) and the fluorescence intensity for Dio-LDL while panel C shows the results from five N342 and five S342 patients (mean intensity fluorescence is given, mean ± SEM is shown).

The relevance of the rs9370867 IDOL SNP on LDL-R expression was further investigated in macrophages generated from peripheral blood mononuclear cells from N342 or S342 IDOL carriers. LDL-R expression and the capacity to internalize LDL particle, as a functional readout of the LDL-R activity. LDL-R mRNA expression and IDOL mRNA expression were similar in N342 and S342 macrophages **(Fig 3A)** and more importantly the genotype was not associated with differences in LDLR function. N342 and S342 macrophages showed indeed the same levels of LDL uptake, as assessed by flow cytometry after incubation of the cells with fluorescently labelled LDL particle **(Fig 3B and Fig 3C)**.

## Discussion

GWAS study in European descent, showed that MYLIP (IDOL) genetic loci are associated with plasma lipid levels [10–12]. The rs9370867 variant on the IDOL gene was initially proposed as a gain of function variant associated with increased LDL-cholesterol levels [13]; however subsequent papers questioned the association of this variant with increased plasma total cholesterol levels both in Brazilians [14] and in Caucasians [15].

For this reason, we investigated in a large cohort from the Italian general population the impact of the same variant of the IDOL gene on plasma lipid parameters and on marker of peripheral atherosclerosis. In agreement with studies in Brazilian and Dutch cohorts [14,15], the IDOL SNP was not associated with any relevant difference on plasma lipid profiles. Of note, while in Europeans the rs9370867 SNP variant is in strong linkage disequilibrium with two upstream genome-wide significant GWAS variants rs2294261 [11], and rs2480 [12], this is not true in the Mexican population [13]. This finding raises the possibility that other SNPs could explain the relevance of the MYLIP locus in the control of plasma lipoprotein levels in Europeans [11] as opposed to what observed in the Mexican population. However neither rs2294261 nor rs2480 exhibited a potential regulatory effect using cis-eQTL analysis [13], suggesting that other studies are need to identify the actual casual variants for these GWAS signals. Furthermore the frequency of allele “A” and “G” alleles for rs9370867 variants of IDOL in our subjects was similar to what reported in larger genome databases but differs from that reported by Weissglas- Volkov et al in the Mexican dyslipidemic cohort [13]. While these aspects support the importance of adjustment for ethnicity, when this was performed in the Brazilian cohort, again no significant association of the N342S variant was observed [14].

In the attempt to investigate whether, despite the absence of a significant association to plasma lipid levels, the rs9370867 variant on the IDOL gene impacts cardiovascular outcome, we investigated its association with cardiac and vascular damage. Carriers of the different alleles for the rs9370867 variant on the IDOL gene present a similar degree of cardiac and vascular dysfunction and more importantly do not differ for the prevalence of either coronary heart disease, peripheral vascular disease or cardiovascular events. Our observations further support a limited impact of this variant in Europeans and strongly support the need of validating the association with cardiovascular outcome also in the Mexican cohort.

Based on the lack of association between the rs9370867 variant with plasma lipid variables, metabolic determinants as well as markers of cardiac and vascular dysfunction, we decided to investigate whether the N342S substitution affects IDOL function. Previous data, generated by cotransfecting human embryonic kidney 293T cells (HEK293T) with plasmids for LDLR and either N342 IDOL or S342 IDOL showed that the presence of N342 is associated with increased LDL-R receptor degradation [13] pointing toward a functional effect for the rs9370867 variant. This finding however was not confirmed in a different work, which was performed under the same experimental conditions by overexpressing the LDL-R and N342S IDOL variant in HEK293T cells [15]. To shed more light on this conflicting result and given that IDOL is widely expressed [7], we decided to investigate directly in primary human cells the impact of the rs9370867 variant and therefore isolated PBMCs and generated macrophages from N342 and from S342 IDOL carriers.

IDOL mRNA expression was not different in N342 or S342 IDOL carriers and more importantly LDL-R functionality, measured as the cell ability to uptake fluorescent labelled LDL was similar between N342 or S342 macrophages. These data obtained in primary cells further limit, also at the molecular levels, the impact of the rs9370867 variant on the IDOL gene. Of note, patients with familial hypercholesterolaemia, when stratified according to the rs9370867 variant, had similar baseline plasma lipid levels but presented a different magnitude of LDL-C reduction following statin therapy, suggesting that a pharmacogenetic effect for this IDOL SNP could exist [29]. A similar finding was observed for another IDOL SNP (Rs6924995) in patients treated with rosuvastatin [30]. In our cohort, NN342 carriers and SS342 carriers, when moved on statin therapy, showed a similar magnitude of LDL-C reduction (data not shown).

Based on these observations, the possibility that, in vitro, sterol deprivation followed by LXR stimulation could maximise the conditions to appreciate a difference, if any, on LDL uptake in carriers of the different isoforms should be considered.

However, it is unlikely, that these conditions occur in the general population and the clinical data point to a similar prevalence of cardiovascular disorders when stratifying for the rs9370867 variant on the IDOL gene.

In conclusion, although IDOL represents a key player in LDL-R biology and subsequent risk of dyslipidemia and atherosclerosis, the functional and clinical impact of the N342S variant is limited. The inclusion of this variant for improving genetic scores for hypercholesterolemia or pharmacological approaches addressed at interfering with the region surrounding the aa 342 of the enzyme should be carefully reconsidered.

## Supporting Information

S1 Table Clinical characteristics according to the rs9370867 SNP in subjects from the PLIC population included in the upper 75^th^ percentile of total cholesterol (A) and triglycerides (B). **-** Multivariate Analysis.
(DOCX)
