# Supplementary material for: IDOL N342S Variant, Atherosclerosis Progression and Cardiovascular Disorders in the Italian General Population
Source: PLoS One. 2015 Apr 30;10(4):e0122414. doi: 10.1371/journal.pone.0122414 (PMC4415795; doi:10.1371/journal.pone.0122414)
Supplement: S1 Table — - Multivariate Analysis. (DOCX) [file pone.0122414.s001.docx]

**S1A and S1B Table. Clinical characteristics according to the rs9370867 SNP in subjects from the PLIC population included in the upper 75^th^ percentile of total cholesterol (A) and triglycerides (B).- Multivariate Analysis**

1. **Upper 75^th^ percentile of Total Cholesterol**

|  | **GG**  **(n= 74)** | **AG**  **(n= 130)** | **AA**  **(n= 72)** | **P-value GG vs AA** | **P-value GG vs GA** | **P-value AA vs GA** |
| --- | --- | --- | --- | --- | --- | --- |
| **Age** | 61.97 ±10.58 | 63.06 ± 10.08 | 64.97 ± 10.22 | 0.293 | 0.708 | 0.207 |
| **Body mass index (Kg/m2)** | 27.2 ± 4.2 | 25.8 ± 3.9 | 26.1 ± 3.3 | 0.198 | 0.076 | 0.340 |
| **Total cholesterol (mg/dL)** | 249.57 ± 21.7 | 249.76 ± 18.58 | 248.90 ± 21.54 | 0.866 | 0.593 | 0.850 |
| **LDL-cholesterol (mg/dL)** | 158.83 ± 25.0 | 161.87 ± 22.16 | 160.20 ± 23.01 | 0.595 | 0.488 | 0.340 |
| **HDL-cholesterol (mg/dL)** | 68.57 ± 19.57 | 65.08 ± 16.04 | 65.63 ± 14.18 | 0.208 | 0.401 | 0.078 |
| **Triglycerides (mg/dL)** | 110.86 ± 47.65 | 114.02 ± 53.71 | 115.38 ± 53.99 | 0.601 | 0.465 | 0.357 |
| **Apolipoprotein A-I (mg/dL)** | 161.41 ± 25.46 | 159.54 ± 22.59 | 158.01 ± 18.38 | 0.101 | 0.094 | 0.060 |
| **Apolipoprotein B (mg/dL)** | 130.88 ± 19.82 | 132.85 ± 18.16 | 131.72 ± 21.18 | 0.525 | 0.809 | 0.265 |
| **Glucose (mg/dL)** | 95.08 ± 12.46 | 93.64 ± 11.78 | 91.35 ± 8.88 | 0.201 | 0.093 | 0.941 |
| **Left Ventricular Mass** | 184.95 ± 64.78 | 156.48 ± 66.93 | 165.74 ± 71.88 | 0.268 | 0.109 | 0.677 |
| **cIMT** | 0.74 ± 0.14 | 0.75 ± 0.17 | 0.77 ± 0.15 | 0.607 | 0.806 | 0.424 |
| **SBP** | 125.0 ± 16.0 | 132.0 ± 20.0 | 126.0 ± 14.0 | 0.076 | **0.036** | 0.107 |
| **DBP** | 78.0 ± 9.0 | 80.0 ± 12.0 | 77.0 ± 7.0 | 0.205 | 0.154 | 0.126 |

**(S1B) Upper 75^th^ Percentile of Triglycerides**

|  | **GG**  **(n=43)** | **AG**  **(n = 81)** | **AA**  **( n= 41)** | **P-value GG vs AA** | | **P-valueGG vs GA** | | **P-valueAA vs GA** |
| --- | --- | --- | --- | --- | --- | --- | --- | --- |
| **Age** | 62.42 ± 10.25 | 64.10 ± 10.55 | 64.33 ± 8.77 | | 0.288 | | 0.575 | 0.563 |
| **Body mass index (Kg/m2)** | 28.0 ± 5.5 | 27.1 ± 4.1 | 28.4 ± 3.9 | | 0.495 | | 0.663 | 0.233 |
| **Total cholesterol (mg/dL)** | 220.53 ± 39.86 | 221.86 ± 35.22 | 224.95 ± 41.25 | | 0.678 | | 0.476 | 0.234 |
| **LDL-cholesterol (mg/dL)** | 134.11 ± 38.80 | 136.27 ± 34.15 | 137.33 ± 40.20 | | 0.987 | | 0.332 | 0.322 |
| **HDL-cholesterol (mg/dL)** | 52.81 ± 11.16 | 49.02 ± 11.34 | 51.07 ± 13.29 | | 0.503 | | 0.525 | 0.167 |
| **Triglycerides (mg/dL)** | 168.05 ± 38.67 | 182.85 ± 55.85 | 182.76 ± 57.24 | | 0.483 | | 0.161 | 0.485 |
| **Apolipoprotein A-I (mg/dL)** | 145.84 ± 19.88 | 142.77 ± 18.69 | 141.83 ± 18.12 | | 0.221 | | 0.591 | 0.434 |
| **Apolipoprotein B (mg/dL)** | 118.42 ± 25.87 | 118.16 ± 24.72 | 119.07 ± 27.01 | | 0.964 | | 0.338 | 0.297 |
| **Glucose (mg/dL)** | 96.77 ± 16.90 | 99.42 ± 26.12 | 100.17 ± 16.21 | | 0.482 | | 0.651 | 0.218 |
| **Left Ventricular Mass** | 190.64 ± 58.76 | 167.56 ± 60.78 | 200.33 ± 84.51 | | 0.494 | | 0.295 | 0.069 |
| **cIMT** | 0.79 ± 0.18 | 0.78 ± 0.19 | 0.76 ± 0.14 | | 0.297 | | 0.433 | 0.745 |
| **SBP** | 130.0 ± 17.0 | 134.0 ± 18.0 | 129.0 ± 21 | | 0.65 | | 0.193 | 0.384 |
| **DBP** | 79.0 ± 70 | 83.0 ± 10.0 | 78.0 ± 6.0 | | 0.81 | | 0.008 | 0.003 |
